# Comment on: Eronen MI (2019). The levels problem in psychopathology

**DOI:** 10.1017/S0033291719003623

**Published:** 2021-02

**Authors:** Harriëtte Riese, Marieke Wichers

**Affiliations:** University of Groningen, Universitair Medisch Centrum Groningen, Department of Psychiatry, Interdisiplinary, Interdisciplinary Center Psychopathology and Emotion regulation, Groningen, The Netherlands

Dear editor,

Recently, Eronen (Eronen, 2019) published a thought provoking philosophical viewpoint to which we would like to add some reflections based on challenges we encountered in the field of (network) research in psychopathology. Eronen argues that in systems with greater complexity, higher levels of explanation may be more useful to find effective interventions. He mentions that the phenomenon of psychopathology is something at a high level of complexity. Therefore, it follows from his reasoning that explanations at the lower level of genes or nervous cells may not be as useful to find effective interventions against psychopathology as higher explanatory levels. We agree with this perspective, and want to further discuss the relevance of this point and add some implications for the field.

In terms of relevance, we think that the notion that the complexity of the studied system influences the optimal level at which we can effectively intervene in the system is of particular importance for psychopathology. In the psychopathology field of research, we aim for knowledge to effectively prevent psychopathology or intervene on existing psychopathology. Indubitably, lower (biological) levels of explanation are involved in psychopathology and can add to the understanding of its mechanisms (Miller, 1996). However, in line with Eronen (2019) we support the idea that the higher psychological level of experienced emotions and behaviour^†^^1^ may be more relevant than the level of genes and cells, or even brain structures, when it comes to finding clues for effective prevention or intervention. This has implications for the optimal focus of research aimed at developing effective interventions. For example, an emergent field is that of network research which studies processes of psychopathology at the level of dynamics between momentary experienced psychological states/behaviour (Borsboom, 2017; Wichers, 2014). An often expressed opinion is that mental disorders, such as psychosis or depression, are *brain* disorders (Insel & Cuthbert, 2015) and that research in this field should thus include biological factors to yield relevant results (Andreasen, 1997; Gordon, 2016). However, in line with prior publications (Borsboom, Cramer, & Kalis, 2019; Eronen, 2013), Eronen (2019) questions the implicit assumption that lower level (biological) explanations are somehow ‘better’ than higher level (psychological) ones. As lower biological levels can be considered permissive to higher level processes (Sapolsky, Romero, & Munck, 2000; Thomas & Sharp, 2019), information at these levels is likely expressed via high levels of experienced psychological states and behaviour. Thus, in the case of network research, we can assume that the dynamics between psychological states and behaviour captures the influence from lower levels. Stated otherwise, we can assume that psychological states and behaviour are, in part, the final expressions of lower level biological processes, which is why network research, for instance, does not necessarily require the inclusion of biological variables. Moreover, it may even be very questionable philosophically to mix different levels of explanation within one (network) model. In fact, it has been pointed out that popular strategies that attempt to link these different levels of explanation are inadequate on logical and conceptual grounds (Miller, 2010; Thomas & Sharp, 2019). Possible future attempts in network research in psychopathology will require intense thought and novel statistical approaches, like the building of, the so-called, multi-layered networks (Boccalettia et al., 2014) as has been used in other scientific fields.

If the assumption is correct that psychological states and behaviour are in part the final expressions of lower level biological processes, then this level has the potential to be very informative. Especially since psychological states and behaviour refer to real-life aspects with which patients present to therapists and which can be targeted during therapy. This higher level of explanation, therefore, may be optimal for finding effective interventions. We can, for example, study individuals with self-monitoring instruments, which provide insights into the precise patterns of these components in the flow of daily life (Myin-Germeys et al., 2018). If we find clues for effective intervention at this level of explanation, then this is easily translated to the precise aspects in an individual's life that may be modifiable, thereby contributing to improvement in precision diagnostics and treatment.

To summarize, even though biology plays an important role in the development and course of psychopathology, studying psychopathology at the level of psychological states and behaviour may yield meaningful outcomes also when biological variables are not included. Moreover, for research aimed at finding effective and personalized interventions, studying psychopathology at this higher level of explanation may be a very relevant and promising approach for the future.

## Footnotes

† The notes appear after the main text.

## Note

1 Although we are aware that besides lower biological processes also the environment or (social) contexts (e.g. Marr, 1982; Thomas & Sharp, 2019) partly influence the processes at the psychological and behavioural level; for the line of argumentation in the manuscript, we have chosen to focus on the lower level.
